# Bone scintigraphy and positron emission tomography in the early diagnosis of MRONJ

**DOI:** 10.1515/med-2025-1143

**Published:** 2025-02-18

**Authors:** Daniele Pergolini, Mohamed Mohsen, Gianluca Tenore, Gaspare Palaia, Lorenzo Magnifico, Alessandro Del Vecchio, Umberto Romeo

**Affiliations:** Department of Oral and Maxillofacial Sciences, Sapienza University of Rome, Rome, Italy; Department of Oral and Maxillofacial Sciences, Sapienza University of Rome, Via Caserta 6, 00161, Rome, Italy

**Keywords:** osteonecrosis of the jaws, bone scintigraphy, positron emission tomography, bisphosphonates, MRONJ, diagnostic techniques

## Abstract

**Objectives:**

The aim of this study is to evaluate the bone scintigraphy (BS) and positron emission tomography (PET) in the early diagnosis of medication-related osteonecrosis of the jaws (MRONJ) and their possible use in the identification of patients at risk for MRONJ.

**Material and methods:**

Thirty-one patients treated with ONJ-related drugs and who had undergone BS or PET for the evaluation of bone lesions were included in the study. The jaws of each patient were divided into four areas. For each area, the presence of pathological tracer uptake was evaluated and related to the eventual MRONJ development. Sensitivity, specificity, and predictive values of both techniques were determined. The latency from the finding of pathological tracer uptake in BS or PET to the clinical diagnosis of MRONJ and the odds ratio were also calculated.

**Results:**

Sensitivity and specificity of BS for MRONJ prediction were, respectively, 83.3 and 87.5%. Positive and negative predictive values were, respectively, 73.2 and 92.8%. The odds ratio was 35. Sensitivity of PET was 33.3%, specificity was 94.9%, and positive and negative predictive values were 70.0 and 80.0%, respectively. The odds ratio was 9.333. All values were statistically significant.

**Conclusions:**

BS and PET may be accurate techniques for an early prediction of MRONJ.

## 1 Introduction

Medication-related osteonecrosis of the jaws (MRONJ) is an adverse drug reaction characterized by the progressive destruction and necrosis of the bone in patients treated with drugs for which an increased risk of MRONJ has been described [1,2]. These drugs include antiresorptive medications such as bisphosphonates and denosumab (a monoclonal antibody), but also some antiangiogenic drugs and immune modulators [3]. Antiresorptive drugs suppress with different mechanisms of the activity of osteoclasts, reducing the risks of skeletal complications in patients with bone loss. For this reason, they are used in patients affected by osteoporosis, bone metastasis from solid tumor, multiple myeloma, and other conditions such as Paget’s disease of bone or giant cell tumor of the bone [4]. Antiresorptive drugs significantly reduce the risk of fracture or other bone complications for these patients [5–9], but their use is associated with the possible development of MRONJ.

**Figure 1:**
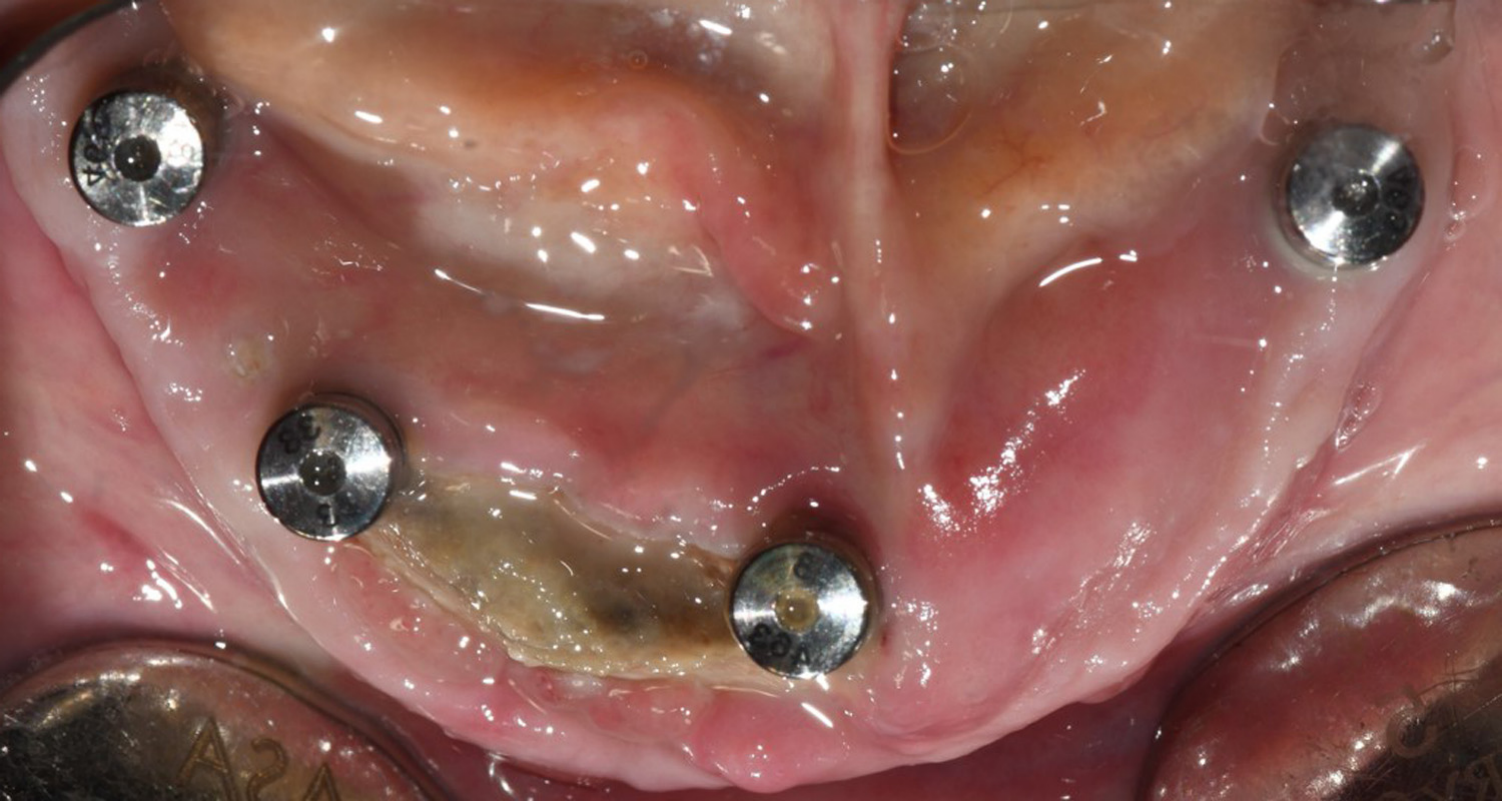
Osteonecrosis of the jaws arising on osteointegrated two implants 6 months after the administration of antiresorptive drugs.

MRONJ is a relatively rare disease and estimates of its incidence are often variable and not accurate. However, different studies agree that incidence and prevalence of MRONJ in oncologic patients are higher than in the osteoporotic ones. The incidence of MRONJ in osteoporotic patients ranges from 1.04 to 69 per 100,000 patient-years, whereas in oncologic patients it ranges from 0 to 90 per 100,000 patient-years [10]. According to the Italian position paper on MRONJ, cancer patients with bone metastasis or multiple myeloma taking antiresorptive drugs present an incidence of MRONJ ranging between 1 and 20%. The risk of MRONJ in osteoporotic patients, instead, is below 1% [11]. These differences are due to the higher dose and different route of administrations (usually intravenous) requested for subjects affected by bone metastasis or multiple myeloma [3,10,12] (Figure 1).

MRONJ can also considerably reduce the quality of life of cancer patients, stressing the importance of a correct and early diagnosis for an optimum treatment [13,14]. MRONJ is a clinical diagnosis based on the presence of exposed bone in the maxillofacial region of patients with current or previous treatment with ONJ-related drugs [3]. Radiographic features of MRONJ are relatively nonspecific. However, different imaging modalities can be useful as an adjunctive aid in the diagnosis and evaluation of MRONJ patients. Among them, we can include intraoral radiographs, panoramic radiograph, computed tomography (CT), cone beam computed tomography (CBCT), magnetic resonance imaging (MRI), bone scintigraphy (BS), and positron emission tomography (PET) [10,15]. As regards plain radiographs, CT, CBCT, and MRI, they may be useful in the identification of the disease and in delineating its extent. They may identify nonspecific changes in the bone such as areas of focal sclerosis, areas of osteolysis, thickened lamina dura, reactive periostal bone, and sequestration [10]. However, they are morphologic imaging techniques that can detect only a macroscopic anatomic change.

BS and PET, instead, are functional imaging modalities able to identify areas of altered bone metabolism through an increased tracer uptake, even without an anatomic change. They can detect minimal and subclinical changes in bones, showing a high sensitivity for detecting early disease [10,16]. Both techniques are widely used in oncology, especially in the diagnosis of bone metastasis and in the follow up of their treatment with antiresorptive drugs [17,18]. For these reasons, cancer patients at high risk of developing MRONJ often possess BS and/or PET that may show an alteration of the jaws, thus helping in an early diagnosis of MRONJ.

The aim of this study is to evaluate the effective role of BS and PET in the early diagnosis of MRONJ and their possible use in the identification of patients at risk for MRONJ development.

## 2 Materials and methods

To address the aim of the study, a retrospective cohort study design was used. To enroll patients, a research was conducted in the electronic database of “Momax” (Oral Medicine and Maxillofacial) project of the Department of Oral Sciences and Maxillofacial Surgery. Inclusion criteria consisted of adult patients with the following features:

1. medical examination performed at the “Policlinico Umberto I” of Rome by an oral pathology specialist in a period ranging from January 2015 to February 2023;
2. previous or ongoing treatment with ONJ-related drugs;
3. BS and/or PET performed to evaluate bone lesions caused by multiple myeloma or bone metastases from solid tumors.

All patients who had performed nuclear medicine imaging studies solely before the beginning of therapy with ONJ-related drugs or after the clinical diagnosis of MRONJ were excluded. Exclusion criteria consisted also of patients with previous treatment with radiotherapy of the head-neck district and with insufficient follow-up to evaluate MRONJ development.

Medical records filled after specialistic examination of all patients included in the study were examined. All data regarding sex, age at first visit, underlying disease who led to the beginning of therapy with ONJ-related drugs, drug administration, date of execution of PET or BS, date of clinical diagnosis of MRONJ, and MRONJ staging according to the AAOMS system [3] were collected.

Whole-body BS was performed approximately 2 h after injection of Technetium-99m bound to methylene or hydroxymethylene diphosphonate. Radiotracer uptake was detected using a single-head gamma camera. As regards PET, it was performed 1 h after injection of fluorodeoxyglucose (^18^F FDG). A high-resolution PET scanner which also incorporated a CT scanner was used. The jaws of each patient were divided into four areas (right maxillary, left maxillary, left mandible, and right mandible). A specialist in nuclear medicine with 15 years of experience and blinded to MRONJ development reviewed BS and PET for pathological tracer uptake. Visual interpretation was performed and the findings were related to the eventual development of MRONJ. If a patient had performed multiple BS or PET, the older scan with a pathological uptake was analyzed. When nuclear medicine imaging techniques did not show any pathological uptake, the most recent scan preceding the clinical diagnosis of MRONJ was used.

True positives, true negatives, false positives, and false negatives were determined and used to calculate sensitivity, specificity, positive, and negative predictive values. The odds ratio and the latency from the finding of pathological tracer uptake in BS or PET to the clinical diagnosis of MRONJ were also calculated. Statistical analysis was conducted using GraphPad Prism (version 9.5.1 for Windows, GraphPad Software, San Diego, California USA, www.graphpad.com). All values were determined with a 95% confidence level. As regards BS, the statistical significance of the sample was calculated by Yates’s chi-squared test, whereas Fisher’s exact test was used for PET. A *p*-value less than 0.05 was considered statistically significant.


**Informed consent:** Informed consent was obtained according to Helsinki Declaration.
**Ethical approval:** The study was approved by the Council of the Department of Oral Sciences and Maxillofacial Surgery at “Sapienza” University of Rome (Prot. N.5 of 20/02/2024).

## 3 Results

A total of 51 patients fulfilled the inclusion criteria. Thirty-one patients had performed BS and 20 patients had performed PET. The median age of the 31 patients who had performed BS was 67.35 years. They were 11 males and 20 females. All of them had taken ONJ-related drugs in order to treat bone metastasis from a solid tumor. The most frequent tumor was breast cancer (64.5% of the patients), followed by prostate cancer (19.4%), lung cancer (9.7%), and kidney cancer (6.4%). Around 14 patients (45.2%) received denosumab by injection, 11 patients (35.5%) received zoledronate intravenously, and 6 patients (19.3%) received both denosumab and zoledronate at different times.

A total of 124 areas were reviewed. Forty-one regions showed pathological tracer uptake. Among them, 30 areas developed MRONJ whereas 11 areas did not develop osteonecrosis despite the positive BS. Among the 83 areas without pathological tracer uptake, only 6 sites developed MRONJ. MRONJ stage 2 was diagnosed in 21 of 31 patients (67.7%), MRONJ stage 3 in 5 patients (16.1%), and MRONJ stage 1 in 3 patients (9.7%). One patient (3.2%) developed MRONJ stage 0 that evolved into stage 1 during the follow up of the disease. Another patient did not develop MRONJ despite the positive BS (Table 1).

**Table 1:** Summary data of BS

	MRONJ development	No MRONJ development	TOT
Positive BS	30	11	41
Negative BS	6	77	83
TOT	36	88	124

Sensitivity and specificity of BS for MRONJ prediction were, respectively, 83.3 and 87.5%. Positive and negative predictive values were, respectively, 73.2 and 92.8%. The odds ratio was 35. Yates’s chi-squared test showed the statistical significance of the sample (*p* < 0.0001). The median latency from the finding of pathological tracer uptake in BS to the clinical diagnosis of MRONJ was 15.96 months with a standard deviation of 11.04 months, showing a great variability.

Twenty patients (5 males and 15 females) with a median age of 68.15 years had performed PET. They took ONJ-related drugs in order to treat bone metastasis from a solid tumor or multiple myeloma. Even in this case the most frequent tumor was breast cancer (60.0% of the patients), followed by lung cancer (20.0%). The remaining patients showed more heterogeneity: one patient (5%) had multiple myeloma, one patient (5%) had prostate cancer, and two patients (10%) were affected by tumors that more rarely spread to the bone (bladder cancer and a rare form of angiosarcoma). Fifteen patients (75.0%) received denosumab by injection, three patients (15.0%) received zoledronate intravenously, and two patients (10.0%) received both denosumab and zoledronate at different times.

A total of 80 areas were reviewed. Ten regions showed pathological tracer uptake. Among them, seven areas developed MRONJ. Among the 70 areas without pathological tracer uptake, 14 sites developed MRONJ. MRONJ stage 1 was diagnosed in 12 of 20 patients (60.0%), MRONJ stage 2 in 4 patients (20.0%), and MRONJ stage 3 in 2 patients (10.0%). One patient (5.0%) did not develop MRONJ despite the positive PET. Another patient did not show pathological tracer uptake and did not develop MRONJ during the follow up (Table 2).

**Table 2:** Summary data of PET

	MRONJ development	No MRONJ development	TOT
Positive PET	7	3	10
Negative PET	14	56	70
TOT	21	59	80

Sensitivity of PET was 33.3%, specificity was 94.9%, and positive and negative predictive values were 70.0 and 80.0%, respectively. The odds ratio was 9.333. Fisher’s exact test showed the statistical significance of the sample (*p* = 0.0025). The median latency from the finding of pathological tracer uptake in PET to the clinical diagnosis of MRONJ was 6.83 months with a standard deviation of 6.49 months. Even in this case there was a great variability.

## 4 Discussion

**Figure 2:**
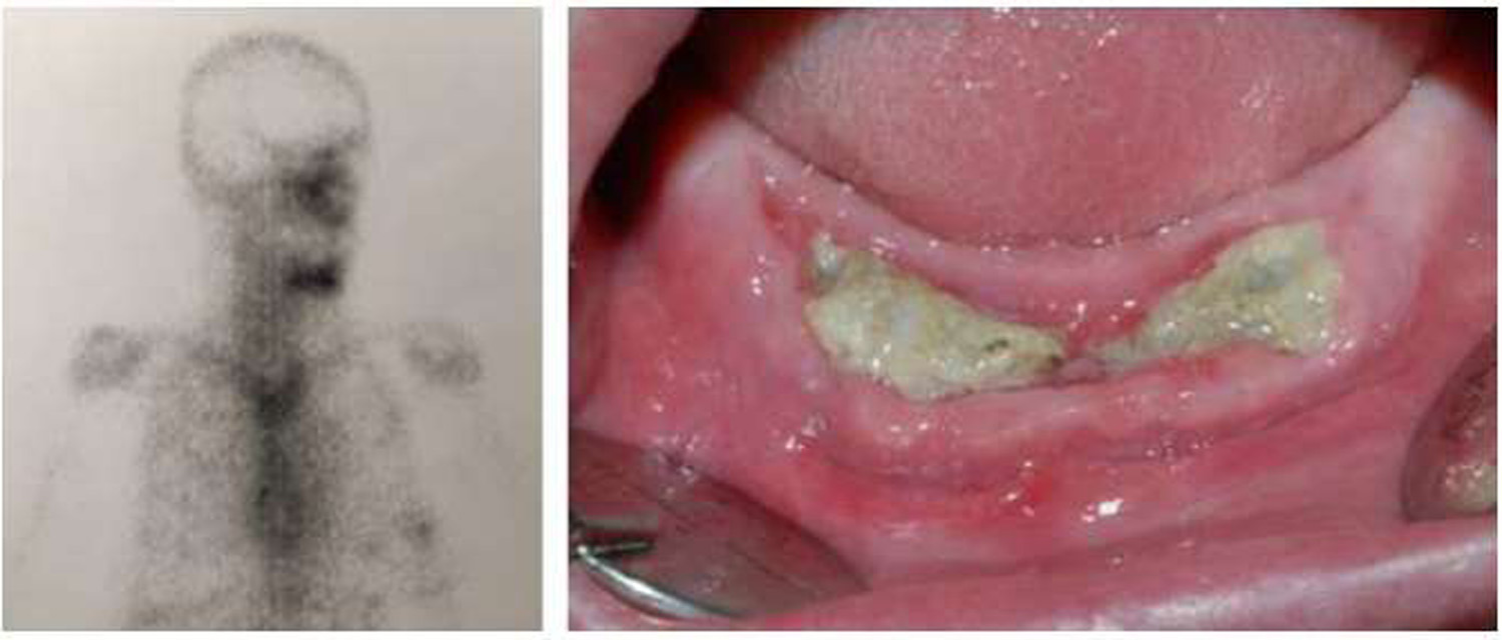
Detail of BS that shows a pathological tracer uptake in areas that developed MRONJ 2 months after the BS was performed.

Different authors proved that BS is able to identify cases of already diagnosed MRONJ as areas with an increased uptake of Technetium-99m [19,20]. Other authors focused on the possible use of pathological tracer uptake as a sign for an early diagnosis of MRONJ. Lesclous et al. reported that strong radionuclide uptake was detected in 5 of 14 patients several months before clinical diagnosis of MRONJ. In the other nine patients, the increased uptake followed tooth extraction [21]. O’Ryan et al. identified 59 cases of intravenous MRONJ. Of the 35 patients who had performed BS before development of MRONJ, 23 (65.7%) had positive tracer uptake in areas that developed MRONJ during the follow up. The latency from the pathological tracer uptake to the clinical diagnosis of MRONJ was 14.4 months on average [22]. Thomas et al. studied 30 patients with metastatic prostate cancer treated with bisphosphonates. Their BS performed after the beginning of the therapy was evaluated for pathological tracer uptake of the jaws and the results were compared to development of MRONJ. In this way, they calculated sensitivity (67%), specificity (79%), positive (44%), and negative (91%) predictive values of BS in the early diagnosis of MRONJ [23]. The results of this study confirm the data reported in the literature. BS showed high sensitivity (83.3%) and specificity (87.5%) in the early diagnosis of MRONJ, with a good positive predictive value (73.2%) and optimum negative predictive value (92.8%). The risk of developing MRONJ was 35-fold increased when BS was positive (Figure 2).

PET can be used as a substitute for BS in the evaluation of bone metastasis. Different authors demonstrated that the technique is useful for detecting areas of altered bone metabolism, allowing to identify cases of already diagnosed MRONJ [24–26]. A very low number of studies focus on the use of PET before the clinical diagnosis of MRONJ and on its use for an early diagnosis. Fujimoto et al. investigated a total of 11 patients who had undergone ^18^F-FDG PET/CT scan over 6 months before diagnosing MRONJ, establishing a protocol that is very similar to the one of the present study. They found good values of sensitivity (80.0%), specificity (72.4%), positive (60.0%), and negative (87.5%) predictive values [27]. Watanabe et al. evaluated a total of 53 patients who underwent ^18^F-FDG PET/CT for oncologic surveillance. Twelve of the 53 patients developed MRONJ after PET was performed. Analyzing the tracer uptake in the jaws, the authors described a higher standardized uptake value in MRONJ patients compared to the control group. Moreover, they calculated sensitivity (75%) and specificity (98%) of PET [28]. Belcher et al., instead, performed a retrospective chart review of 20 patients whose PET scans were acquired within 1 year of MRONJ diagnosis. They found diametrically opposite results, determining low values of sensitivity (43%) and specificity (19%) [29] (Tables 3 and 4).

**Table 3:** Summary data of patients with BS

#Patient	Sex	Age	Pathology	Medication	Latency from pathological tracer uptake to clinical diagnosis	MRONJ stage
1	M	87	Prostate cancer	Zoledronate	2 months	2
2	M	73	Kidney cancer	Zoledronate	20 months	2
3	F	71	Breast cancer	Zoledronate and denosumab	14 months	2
4	M	76	Prostate cancer	Zoledronate	No uptake	2
5	F	63	Breast cancer	Denosumab	No uptake	2
6	M	73	Lung cancer	Denosumab	9 months	1
7	F	85	Breast cancer	Denosumab	2 months	2
8	F	70	Breast cancer	Zoledronate	No uptake	3
9	F	76	Breast cancer	Zoledronate	0 months	2
10	F	61	Breast cancer	Zoledronate	1 month	3
11	F	53	Breast cancer	Zoledronate and denosumab	No uptake	1
12	M	76	Prostate cancer	Zoledronate	6 months	2
13	F	57	Breast cancer	Denosumab	24 months	1
14	F	54	Breast cancer	Denosumab	24 months	2
15	F	74	Breast cancer	Denosumab	No MRONJ	NO
16	F	58	Breast cancer	Zoledronate	27 months	2
17	F	57	Breast cancer	Zoledronate and denosumab	7 months	2
18	F	64	Breast cancer	Denosumab	1 month	3
19	F	62	Breast cancer	Denosumab	12 months	2
20	M	84	Lung cancer	Denosumab	29 months	3
21	M	74	Lung cancer	Denosumab	13 months	3
22	M	62	Prostate cancer	Denosumab	21 months	2
23	M	62	Prostate cancer	Denosumab	23 months	2
24	F	61	Breast cancer	Zoledronate and denosumab	11 months	2
25	M	75	Prostate cancer	Denosumab	No uptake	2
26	F	66	Breast cancer	Zoledronate and denosumab	28 months	2
27	F	54	Breast cancer	Zoledronate and denosumab	12 months	2
28	F	68	Breast cancer	Denosumab	22 months	0
29	F	63	Breast cancer	Zoledronate	20 months	2
30	F	61	Breast cancer	Zoledronate	41 months	2
31	M	68	Kidney cancer	Zoledronate	30 months	2

**Table 4:** Summary data of 31 patients with BS

Sex	11 males (35.5%) and 20 females (64.5%)
Median age	67.35 years
Pathology	20 breast cancer (64.5%), 6 prostate cancer (19.4%), 3 lung cancer (9.7%), and 2 kidney cancer (6.4%)
Medication	14 denosumab (45.2%), 11 zoledronate (35.5%), 6 both denosumab and zoledronate (19.3%)
Median latency from pathological tracer uptake to clinical diagnosis	15.96 months
MRONJ stage	21 stage 2 (67.7%), 5 stage 3 (16.1%), 3 stage 1 (3.2%), 1 stage 0 (9.7%), 1 no MRONJ (3.2%)

Our results regarding PET, although preliminary, are encouraging. We obtained good positive (70.0%) and negative (80.0%) values and a really high specificity (94.9%). The risk of developing MRONJ was 9.333-fold increased when PET was positive. Quite the opposite, sensitivity was low (33.3%), because different areas who developed MRONJ had negative PET scans. This result could be explained by different factors, such as the variable and high amount of time from the acquisition of the scan to the diagnosis of MRONJ. Moreover, the uptake of ^18^F-FDG seems to occur only in areas where osteonecrosis is associated with an inflammatory-infectious process, so the initial stages of MRONJ with low inflammatory component could result negative [30–32] (Tables 5 and 6).

**Table 5:** Summary data of patients with PET

#Patient	Sex	Age	Pathology	Medication	Latency from pathological tracer uptake to clinical diagnosis	MRONJ stage
1	M	65	Lung cancer	Denosumab	No uptake	1
2	M	52	Lung cancer	Denosumab	No uptake and no MRONJ	NO
3	F	62	Breast cancer	Denosumab	1 month	2
4	F	69	Multiple myeloma	Zoledronate	No uptake	1
5	M	69	Bladder cancer	Denosumab	5 months	2
6	F	76	Breast cancer	Denosumab	No uptake	2
7	F	77	Breast cancer	Denosumab	No uptake	1
8	F	58	Lung cancer	Denosumab	No uptake	1
9	F	70	Breast cancer	Zoledronate and denosumab	No uptake	3
10	F	73	Breast cancer	Denosumab	No uptake	1
11	F	76	Breast cancer	Denosumab	11 months	1
12	F	67	Breast cancer	Zoledronate	2 months	1
13	M	66	Prostate cancer	Zoledronate and denosumab	No uptake	3
14	F	71	Breast cancer	Denosumab	4 months	1
15	F	59	Breast cancer	Denosumab	No uptake	1
16	F	58	Angiosarcoma	Denosumab	No MRONJ	NO
17	F	75	Breast cancer	Denosumab	No uptake	2
18	F	78	Breast cancer	Denosumab	18 months	1
19	F	69	Breast cancer	Zoledronate	No uptake	1
20	M	73	Lung cancer	Denosumab	No uptake	1

**Table 6:** Summary data of 20 patients with PET

Sex	5 males (25%) and 15 females (75%)
Median age	68.15 years
Pathology	12 breast cancer (60.0%), 4 lung cancer (20.0%), 1 multiple myeloma (5.0%), 1 prostate cancer (5.0%), 2 tumors that rarely spread to the bone (10.0%)
Medication	15 denosumab (75.0%), 3 zoledronate (15.0%), 2 both denosumab and zoledronate (10.0%)
Median latency from pathological tracer uptake to clinical diagnosis	6.83 months
MRONJ stage	12 stage 1 (60.0%), 4 stage 2 (20.0%), 2 stage 3 (10.0%), 2 no MRONJ (10.0%)

Despite the promising results, there are several limitations of this study. Because of the low prevalence of MRONJ, a relatively smart number of patients, especially who had performed PET, were included. The retrospective nature of this study also causes a high variability of the median latency from the finding of pathological tracer uptake in BS or PET to the clinical diagnosis of MRONJ, whose results should be considered with caution. A prospective study is needed to allow a better assessment. Furthermore, giving the specialistic nature of “Momax” project, the oral health status of the patients prior to our first visit and MRONJ diagnosis was often unknown. For this reason, the possibility that the positive uptake reflected dental or periodontal disease preceding MRONJ development cannot be excluded.

## 5 Conclusions

BS and PET can identify bone alterations earlier and with more precision compared to traditional radiological procedures. They are used to identify and to assess response to treatment of bone metastasis in cancer patients, which are also at high risk of developing MRONJ. Representing the whole body of the patients, they could discover pathological alterations of the jaws and allow an early diagnosis of MRONJ. Despite the limitations, this study shows that BS and PET with pathological tracer uptake in the jaws are associated with an increased risk of developing MRONJ. On the contrary, if the exams are negative development of MRONJ in the future has a low probability. For these reasons, they may be accurate techniques for an early prediction of MRONJ. For daily clinical practice, our results suggest nuclear medicine specialists to search for pathological tracer uptake in the jaws when they examine BS or PET of patients taking ONJ-related drugs. Moreover, the clinician should also ask the patient if he has performed BS or PET in the past, in order to use them as a valid aid in the early diagnosis of MRONJ.
